# Association between vessel-specific coronary Aggregated plaque burden, Agatston score and hemodynamic significance of coronary disease (The CAPTivAte study)

**DOI:** 10.1016/j.ijcha.2024.101384

**Published:** 2024-03-10

**Authors:** Avedis Ekmejian, Nicklas Howden, April Eipper, Usaid Allahwala, Michael Ward, Ravinay Bhindi

**Affiliations:** a Royal North Shore Hospital, Australia; b North Shore Private Hospital, Australia; c University of Sydney Northern Clinical School, Australia

**Keywords:** FFR, CTCA, APB, Plaque Burden, Plaque Volume, AI

## Abstract

**Background:**

CT coronary angiography (CTCA) is a guideline-endorsed assessment for patients with stable angina and suspected coronary disease. Although associated with excellent negative predictive value in ruling out obstructive coronary disease, there are limitations in the ability of CTCA to predict hemodynamically significant coronary disease. The CAPTivAte study aims to assess the utility of Aggregated Plaque Burden (APB) in predicting ischemia based on Fractional Flow Reserve (FFR).

**Methods:**

In this retrospective study, patients who had a CTCA and invasive FFR of the LAD were included. The entire length of the LAD was analyzed using semi-automated software which characterized total plaque burden and plaque morphological subtype (including Low Attenuation Plaque (LAP), Non-calcific plaque (NCP) and Calcific Plaque (CP). Aggregated Plaque Burden (APB) was calculated. Univariate and multivariate analysis were performed to assess the association between these CT-derived parameters and invasive FFR.

**Results:**

There were 145 patients included in this study. 84.8 % of patients were referred with stable angina. There was a significant linear association between APB and FFR in both univariate and multivariate analysis (Adjusted R-squared = 0.0469; p = 0.035). Mean Agatston scores are higher in FFR positive vessels compared to FFR negative vessels (371.6 (±443.8) vs 251.9 (±283.5, p = 0.0493).

**Conclusion:**

CTCA-derived APB is a reliable predictor of ischemia assessed using invasive FFR and may aid clinicians in rationalizing invasive vs non-invasive management strategies. Vessel-specific Agatston scores are significantly higher in FFR-positive vessels than in FFR-negative vessels. Associations between HU-derived plaque subtype and invasive FFR were inconclusive in this study.

## 1 Introduction

Stable angina represents a substantial disease burden across the globe, with angina affecting 10.8 million people in the United States alone [1]. Recent evidence has demonstrated that non-invasive strategies for the management of stable angina reduces the incidence of Major Adverse Cardiovascular Events (MACE), using Computed Tomography Coronary Angiography (CTCA) to risk stratify patients with stable angina [2]. Reducing the reliance on invasive techniques has the additional benefit of cost efficiency [3]. It is acknowledged that there are multiple factors which contribute to the hemodynamic burden of coronary disease [4], and that relying purely on the luminal assessment of stenosis may result in the misclassification of ischaemic status due to visual-functional mismatch for both invasive angiography and CTCA [5], [6], [7].

Fractional flow reserve (FFR) is a reliable invasive functional assessment of the hemodynamic severity of stable coronary disease and has been demonstrated to reduce the incidence of MACE for patients managed with an invasive strategy, predominantly driven by reduced urgent revascularisation [8]. Recognising that there is a role for both invasive and non-invasive strategies for patients with stable angina, there remains room to refine referral strategies for invasive strategies based on CTCA [9].

CT-FFR, which applies computational fluid dynamics to derive *trans*-stenotic gradients [10], is an emerging field in the non-invasive assessment of ischaemic burden of coronary disease. However, this luminal assessment of does not consider the potential impact of plaque burden and plaque morphology on coronary hemodynamics. Furthermore, accessibility of this technology has limited its widespread utility.

**Fig. 1:**
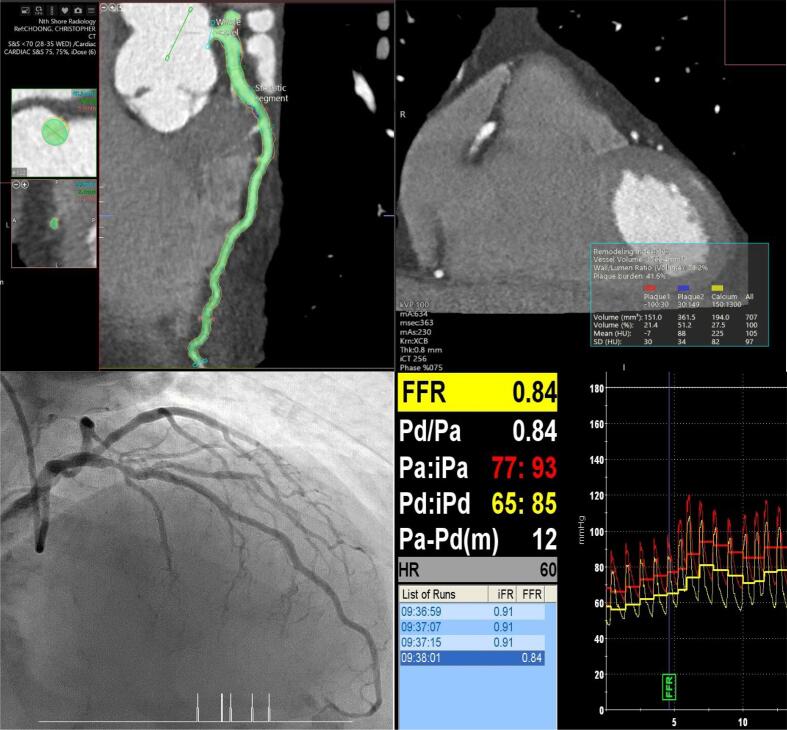
Central Illustration: 145 patients who had a CTCA and invasive FFR of the LAD were included. The entire length of the LAD was analyzed using semi-automated software which characterized total plaque burden and plaque morphological subtype. This study showed an association between CTCA-derived Aggregated Plaque Burden (APB) and invasive FFR.

Accordingly, numerous recent studies have taken advantage of the ability of CTCA to assess plaque morphology and burden, in addition to luminal stenosis, to better predict hemodynamically significant coronary disease, allowing more appropriate referral for invasive coronary angiography (ICA). Specifically, the emergence of Automated Intelligence (CTCA-AI) with deep learning and machine learning [11], has facilitated the accurate quantification and characterisation of plaque, which has provided findings consistent with intra-vascular imaging, including intra-vascular ultrasound (IVUS) [12]. The additional advantage of this modality compared to intra-vascular imaging is the capacity to segment the entire vessel Fig. 1

Studies by Driessen et al [13] and Gaur et al [14] investigated the relationship between vessel plaque burden (for all coronary segments >2 mm) and FFR-derived ischemia, showing clear relationships between plaque burden and FFR. There have been potential improvements in the imaging technology, which allow the visualisation of the entire coronary artery, facilitating plaque quantification of the entire vessel [15]. This is important, as FFR is usually measured in the terminal vessel, which will assess the ischaemic burden of the aggregate of plaque throughout the vessel [16]. Furthermore, FFR has limited capacity to assess the lesion-specific burden of ischemia due to the effect of serial lesions on coronary flow [17].

In the CAPTivAte Study, we sought to investigate the association between aggregated plaque burden (APB) throughout the entire coronary artery, and invasive FFR. Additionally, we aim to investigate the relationship between plaque morphological subtype and FFR.

## 2 Methods

2.1 Study design and rationale

This was a retrospective observational study. The procedural database at two tertiary centres was reviewed for patients who had invasive angiography with subsequent FFR of the LAD between January 2014 and December 2022. The CTCA database was subsequently reviewed for CTCA studies performed within 2 months of the FFR procedure. Cases were excluded if:

– There was significant artefact limiting analysis of the target vessel.
– The vessel had been previously stented or grafted.
– There was incomplete reconstruction of the entire target vessel on CTCA.
– The FFR was not recorded by positioning the pressure wire at least 2 cm distal to the most distal plaque.

Only LAD arteries were analysed for this study. This was to reduce the impact of hydrostatic pressure gradients [18] and myocardial supply area [19] on the FFR readings, which contributes to a statistically significant difference of mean FFR between the three major epicardial arteries [20].

Invasive angiography and coronary physiology Measurements

Angiography and physiology measurements were consistent with standard practice. After administering intra-coronary glyceryl trinitrate (50–200 mcg) and normalising the pressure wire, the wire was inserted at least 2 cm distal to the most distal visible plaque. Hyperaemia was induced by intravenous adenosine (140–180 μg/kg/min) or intra-coronary adenosine bolus (200–400 mcg). FFR ≤0.80 was considered ischaemic.

## 3 CTCA acquisition

CTCA images were acquired using CT scanners with 256–320 detector rows. Beta-blockers were given if required to achieve a target heart rate of <60 beats per minute. Sublingual glyceryl trinitrates were given to all patients prior to scanning.

### 3.1 Coronary plaque analysis and Definitions

The entire length of the coronary artery was analysed using semi-automated attenuation-based software tool (CT SUREPlaque, Vitrea AV Version 7.14.6; Canon Medical Informatics and Canon Medical Systems Corporation). Luminal cross-sections of 1 mm spacing were analysed by experienced CTCA reporters who were blinded to the FFR reading, with each cross-section analysed first by the automated software to identify borders of the external vessel wall, lumen, and plaque. Manual adjustments were made if required on every 1 mm slice. After confirming accurate segmentation of the artery, the SurePlaque software calculated the aggregated plaque volume and maximal area/diameter stenosis. Volumes of plaque subtypes were also obtained through the software, using pre-specified definitions of Low Attenuation Plaque (LAP), Non-Calcific Plaque (NCP) and Calcific Plaque (CP), according to consensus guidelines from SCCT [21] and Intravascular Ultrasound (IVUS) correlation data [22]. LAP was defined as plaque with attenuation <30 Hounsfield units (HU). NCP was defined as attenuation between 30 and 150 HU, and CP was defined as attenuation >150 HU. Aggregated Plaque Burden (APB) was calculated as aggregated plaque volume/vessel volume (APV/VV) × 100 % [23]. Subtypes of plaque burden, including CP burden (CPB), LAP burden (LAPB), and NCP burden (NCPB) were defined as plaque subtype volume as a percentage of overall plaque volume. Volumes of plaque subtypes included CP volume (CPV), LAP volume (LAPV) and NCP volume (NCPV).

The software also provided a Tortuosity Index (TI), which was defined as the ratio between the length of the coronary artery along the centreline and the linear distance between the proximal and distal endpoints [24]. Lesion length was at the site of the maximal area stenosis and measured from the proximal reference area with minimal plaque burden to the distal reference area with minimal plaque burden.

### 3.2 Statistical Analysis

Statistical analyses were performed using STATA software (version 14.0, StataCorp LP). Variables were analysed and presented on a per-vessel basis in terms of frequencies and percentages for categorical variables and mean and standard deviation (SD) for continuous variables. Univariate and multivariate analysis were performed to assess the association between clinical characteristics, CT-derived measurements, and FFR. Pearson correlation coefficient tests were used for a linear correlation between continuous parameters and Spearman correlation coefficient test were used for ordinal parameters. The odds ratios and associated 95 per cent confidence intervals (95 % CI) for variables in the final model were reported. Statistical significance for this model was set for p-value <0.05. Correlations among the predictors included were assessed for collinearity.

The study protocol was approved a priori by the Northern Sydney Local Health District Human Research Ethics Committee (HREC) and conforms to the ethical guidelines of the 1975 Declaration of Helsinki research committee (Study ID – 2022/ETH02756). A waiver of consent was applied due to the retrospective nature of the study.

## 4 Results

There were 145 patients included in this study. Mean age was 67 (±10.7). 84.8 % of patients were referred with stable angina, of which 26.2 % were female. The mean duration between CTCA and invasive FFR was 20.0 (±6.9) days. The baseline characteristics are summarised in Table 1.

**Table 1:** Baseline Characteristics.

Variable N(%)	FFR positive (OR; 95 % CI, p-value)	FFR positive (OR; 95 % CI, p-value)	Mean FFR (±SD)	Mean APB (%)	Mean APV (mm^3^)
	Univariate Analysis	Multivariate Analysis			
Stable Angina 123 (84.8 %)	1.20 (0.47–3.07); p = 0.706	1.56 (0.55–4.41); p = 0.398	0.82 (±0.07) vs 0.81 (±0.07)	52.3 (±8.3) vs 50.8 (±8.3)	925.6 (±343.6) vs 944.0 (±327.0)
Female Gender 38 (26.2 %)	0.37 (0.16–0.85); p = 0.019	0.36 (0.015–0.84); p = 0.019	0.84 (±0.06) vs 0.81 (±0.06)	50.5 (±6.4) vs 52.6 (±6.3)	760.6 (±268.2) 988.0 (±344.8)
Diabetes 18 (12.4 %)	1.56 (0.58–4.22); p = 0.378	1.75 (0.62–4.95); p = 0.292	0.8 (±0.07) vs 0.82 (±0.07)	50.5 (±10.9) vs 52.3 (±7.6)	977.3 (344.1) vs 921.4 (340.3)
Hypertension 87 (60 %)	0.74 (0.37–1.48); p = 0.391	0.92 (0.43–1.98); p = 0.829	0.82 (±0.06) vs 0.81 (±0.06)	52.7 (±8.4) vs 51 (±8.3)	913.6 (±327.7) vs 950.5 (±360.0)
Cholesterol 94 (64.8 %)	0.64 (0.32–1.27); p = 0.202	0.56 (0.24–1.27); p = 0.164	0.82 (±0.07) vs 0.81 (±0.07)	52.4 (±8.0) vs 51.3 (±8.1)	934.6 (±353.1) vs 925 (± 325.1)
Smoking 44 (±30.3 %)	1.08 (±0.53–2.23); p = 0.829	0.95 (±0.44–2.02); p = 0.895	0.81 (±0.07) vs 0.82 (±0.07)	52.6 (±8.0) Vs 51.8 (±7.9)	957.5 (±388.7) vs 915.7 (±384.4)

FFR = Fractional Flow Reserve; APB (%) = Aggregated Plaque Burden; APV = Aggregated Plaque Volume.

### 4.1 Vessel Characteristics

**Fig. 2:**
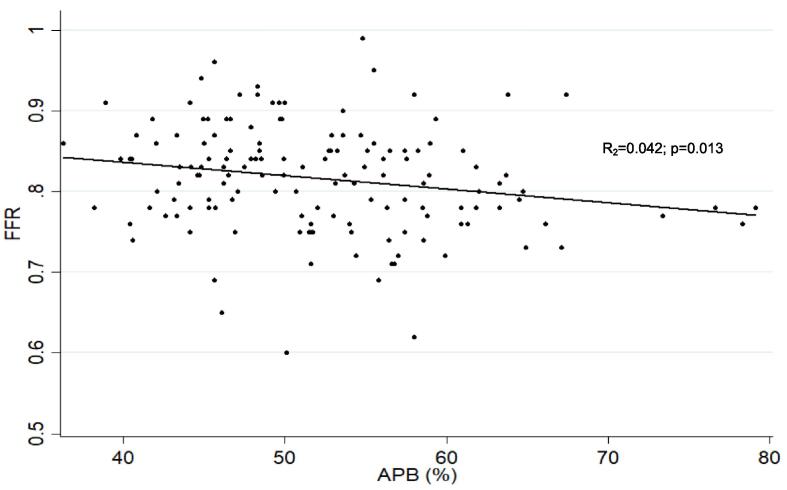
Linear Logistic Regression correlating APB and FFR, showing there is a statistically significant correlation between APB and FFR.

**Fig. 3:**
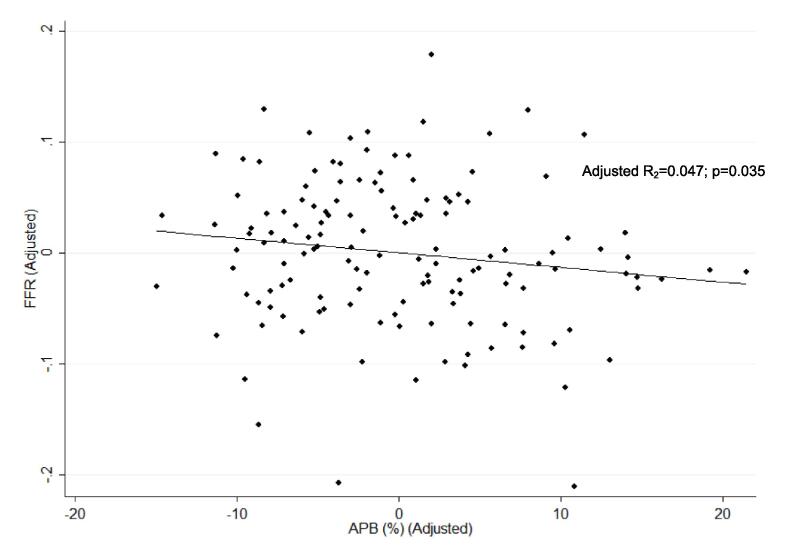
Linear Logistic Regression correlating APB and FFR, adjusted for maximal area stenosis and length of plaque. There is a statistically significant relationship between APB and FFR, independent of maximal area stenosis or length of plaque.

Mean FFR was 0.82 (±0.07). FFR was positive in 58 (40 %) of vessels. 55 (37.9 %) were proximal, 87 (60 %) were in the mid-vessel, and 3 (2.1 %) were in the distal vessel. The OR of positive FFR for proximal vs non-proximal disease was 1.22 (0.60–2.50); p = 0.572, adjusted for plaque burden and maximal area stenosis. Table 2 summarises the relationship between CT-derived vessel characteristics and FFR-derived ischemia, and Table 3 summarises the correlation between plaque volumes by subtype and FFR. Fig. 2 depicts the linear relationship between APB and FFR, and Fig. 3 depicts the linear relationship between APB and FFR after adjusting for maximal area stenosis and plaque length.

**Table 2:** Relationship between CT-derived vessel characteristics and FFR-derived ischemia.

Variable	Mean value (±SD)			p-value
	Overall (±n = 145)	FFR ≤ 0.80 (±n = 58)	FFR > 0.80 (±n = 87)	
VV (mm^3^)	1963.5 (±2044.7)	1840.1 (±512.7)	2045.8 (±2610)	0.5548
APB (%)	52.1 (±8.1)	54.6 (±9.6)	50. (±6.4)	0.0015
APV (mm^3^)	933.4 (±340)	986.8 (±389)	866.4 (±298)	0.0369
CPV (mm^3^)	332.1 (±179.9)	373.7 (±219.2)	304.3 (±144.3)	0.0229
LAPV (mm^3^)	198.6 (±94.7)	202.8 (±95.5)	195.8 (±94.7)	0.6645
NCPV (mm^3^)	390.8 (±141.1)	403 (±129.1)	382.7 (±148.9)	0.3983
CPB (%)	35.7 (±12.3)	36.7 (±8.0)	34.9 (±7.4)	0.167
LAPB (%)	21.4 (±6.9)	20.4 (±4.1)	22.0 (±3.7)	0.0158
NCPB (%)	42.9 (±9.0)	41.9 (±4.9)	43.5 (±4.6)	0.0475
Agatston Score	309.9 (±363.3)	371.6 (±443.8)	251.9 (±283.5)	0.0493
Maximal Area Stenosis (%)	69.3 (±18.8)	71.5 (±20.8)	66.5 (±17.2)	0.1173
Lesion length (mm)	20.6 (±14.9)	22.0 (±15.7)	19.1 (±14.2)	0.2502
RI	1.33 (±0.30)	1.37 (±0.31)	1.31 (±0.30)	0.2463
TI	1.37 (±0.29)	1.32 (±0.31)	1.40 (±0.27)	0.1018

FFR = Fractional Flow Reserve; VV = Vessel Volume; APB (%) = Aggregated Plaque Burden; APV = Aggregated Plaque Volume; CPV = Calcific Plaque Volume; LAPV = Low-Attenuation Plaque Volume; NCPV = Non-Calcific Plaque Volume; CPB (%) = Calcific Plaque Burden; LAPB (%) = Low-Attenuation Plaque Burden; NCPB = Non-Calcific Plaque Burden; RI = Remodelling Index; TI = Tortuosity Index.

**Table 3:** Correlation between volumes of plaque subtypes and FFR.

	Univariate Analysis		Multi-variate analysis*	
Variable	R^2^	p-value	R^2^	p-value
CPV (mm^3^)	0.0194	0.095	0.0282	0.266
NCPV (mm^3^)	0.0020	0.593	0.0205	0.695
LAPV (mm^3^)	0.0007	0.754	0.0209	0.648
CPB (%)	0.0262	0.052	0.0209	0.640
NCPB (%)	0.0248	0.059	0.0205	0.694
LAPB (%)	0.0020	0.59	0.0207	0.662
Agatston Score	0.0203	0.093	0.0305	0.194

CPV = Calcific Plaque Volume; LAPV = Low-Attenuation Plaque Volume; NCPV = Non-Calcific Plaque Volume; CPB (%) = Calcific Plaque Burden; LAPB (%) = Low-Attenuation Plaque Burden; NCPB = Non-Calcific Plaque Burden.

* Adjusted for maximal stenosis and lesion length.

## 5 Discussion

This study demonstrates a significant association between APB and the hemodynamic significance of coronary disease, as assessed by invasive FFR. This finding remains significant even after adjusting for other clinical and CT-derived parameters, emphasizing the potential utility of APB in predicting clinically significant coronary disease. As semi-automated plaque detection improves in accuracy and becomes a fully automated process, there may be an emerging role in quantifying plaque burden as a risk-stratification tool for hemodynamically significant coronary disease and rationalise invasive versus non-invasive management strategies following CTCA.

In our study, the relationships between plaque volume by subtype and FFR were inconsistent depending on the method used to analyse these relationships. There was no statistically significant correlation between plaque volume (mm^3^) and FFR values. Similarly, plaque burden by subtype had no relationship with FFR. Contrary to this, mean plaque burden and mean plaque volume was significantly higher for LAP and NCP when comparing FFR negative to FFR positive lesions. Similarly, the mean Agatston score was higher in FFR positive vessels, however this association was weakly statistically significant, and there was no significant linear relationship between Agatston score and FFR. Therefore, the Agatston score may help in risk stratifying patients, but has less utility in predicting vessel-specific ischemia compared to APB. Overall, we cannot draw firm conclusions regarding the impact of plaque subtype on FFR.

Other studies which using invasive intra-coronary imaging to define plaque subtype, including both IVUS [25] and Optical Coherence Tomography (OCT)[26], have also not identified a relationship between most plaque subtypes and FFR, although lipid-rich plaque or necrotic core did seem to be associated with FFR-derived ischaemia [27]. The study by Gaur et al [14] showed that mean plaque volumes of all 3 subtypes were significantly higher in FFR positive vessels compared to FFR negative.

Although there have been studies validating CT-derived plaque morphology using HU thresholds, there is likely to be some misclassification of plaque morphology and mis-quantification of the plaque burden by applying these binary thresholds, which may in part contribute to the lack of association between plaque morphology and FFR seen in this study. Moreover, HU-defined plaque subtypes can be affected by the attenuation of the coronary lumen as demonstrated by Maffei et al [28]. Advanced AI algorithms may facilitate more accurate designation of plaque subtype. Iterative Model Reconstruction (IMR) algorithms [29] designed to improve contrast-to-noise ratios, also been applied experimentally to assess plaque morphology and plaque volumes. These algorithms have resulted in superior correlation between CT-derived plaque volumes and IVUS for all plaque subtypes when compared to more traditional filtered back projection (FBP) algorithms [30]. Calcific plaque is particularly problematic in assessing plaque volumes due to blooming artefact. De-blooming algorithms been trialled by Li et al [31], showing promise in improving plaque quantification and luminal stenosis estimation.

Our study found that female gender was a predictor of negative FFR, and mean FFR was significantly higher in the female subgroup compared to male. This is consistent with multiple other studies, including a sub-study of the FAME study [32]. The mechanism for this observation may relate to relative attenuation of coronary flow in the hyperaemic state. A study by Chung et al [33] has shown no difference in microvascular function between men and women, however Coronary Flow Reserve (CFR) was lower for women than men, due to higher resting coronary flow.

In our study we found that tortuosity, measured using TI, did not appear to be associated with FFR. Computational Fluid Dynamic (CFD) modelling predicts that tortuosity reduces coronary perfusion pressures [34], because of inefficient helical flow generated by the curvature of the vessel. This has not been demonstrated in vivo to date [26]. It is possible this study failed to demonstrate an association due to the method the TI was calculated. This distal vessel is likely to be more tortuous, and therefore contribute disproportionately to TI compared to its myocardial supply area. Furthermore, instrumentation of the coronary using a wire may confound the true impact of tortuosity on FFR by straightening the vessel. Lastly, tortuosity is associated with various left ventricular phenotypes, including one study showing a reduced left ventricular mass for tortuous vessels with relative longitudinal shortening of the LV [35]. Lower LV mass results in less coronary flow, as myocardial supply area is an important determinant of coronary flow, and this will subsequently reduce the pressure gradient across the lesion in FFR [19].

### 5.1 Limitations

Although there are clinically relevant observations that may be drawn from this research, there are several limitations. One notable limitation of this study was the small sample size of 145 vessels. Although this sample size was sufficient to detect a statistically significant interaction between APB and invasive FFR, the impact of plaque subtypes on invasive FFR could not reliably be assessed with this limited sample size. A larger prospective data-set will be required to establish the APB threshold associated with ischaemia and adverse clinical outcomes. The retrospective nature of this study has resulted in a temporal gap between CTCA and invasive FFR, with potential changes in plaque composition, although limiting the time temporal gap to 2 months is unlikely to result in any significant progress of disease in the stable setting, particularly with a mean duration of 20 days. Additionally, the retrospective nature of this study has resulted in a potential bias with only patients with moderate to severe disease on CTCA referred for invasive angiography. Therefore, the results may not be generalisable to the minor to moderate spectrum of coronary disease. Furthermore, this study focused on the LAD, therefore the generalisation of findings to other coronary arteries requires further investigation. Lastly, despite the strengths of automated software in vessel segmentation, manual adjustment was still necessary for accurate plaque quantification and assessment of luminal stenosis. Future research should focus on improving the accuracy and automation of lumen and plaque detection software to improve its utility in clinical practice.

## 6 Conclusions

CTCA-derived APB is a reliable predictor of ischemia assessed using invasive FFR and may aid clinicians in rationalising invasive vs non-invasive management strategies. Vessel-specific Agatston scores are significantly higher in FFR positive vessels than FFR negative vessels. Associations between HU-derived plaque subtype and invasive FFR were inconclusive in this study.

## CRediT authorship contribution statement

**Avedis Ekmejian:** Writing – review & editing, Writing – original draft, Visualization, Resources, Methodology, Investigation, Formal analysis, Data curation, Conceptualization. **Nicklas Howden:** Writing – review & editing, Methodology, Data curation. **April Eipper:** Data curation. **Usaid Allahwala:** Writing – review & editing, Conceptualization. **Michael Ward:** Writing – review & editing, Conceptualization. **Ravinay Bhindi:** Writing – review & editing, Supervision, Conceptualization.

## Declaration of competing interest

The authors declare that they have no known competing financial interests or personal relationships that could have appeared to influence the work reported in this paper.
